# *Candida albicans AGE3*, the Ortholog of the *S. cerevisiae* ARF-GAP-Encoding Gene *GCS1*, Is Required for Hyphal Growth and Drug Resistance

**DOI:** 10.1371/journal.pone.0011993

**Published:** 2010-08-05

**Authors:** Thomas Lettner, Ute Zeidler, Mario Gimona, Michael Hauser, Michael Breitenbach, Arnold Bito

**Affiliations:** 1 Department of Cell Biology, University of Salzburg, Salzburg, Austria; 2 Institut Pasteur, Unité Biologie et Pathogénicité Fongiques, Paris, France; 3 Department of Molecular Biology, University of Salzburg, Salzburg, Austria; CNRS UMR6543, Université de Nice, Sophia Antipolis, France

## Abstract

**Background:**

Hyphal growth and multidrug resistance of *C. albicans* are important features for virulence and antifungal therapy of this pathogenic fungus.

**Methodology/Principal Findings:**

Here we show by phenotypic complementation analysis that the *C. albicans* gene *AGE3* is the functional ortholog of the yeast ARF-GAP-encoding gene *GCS1*. The finding that the gene is required for efficient endocytosis points to an important functional role of Age3p in endosomal compartments. Most *C. albicans age3*Δ mutant cells which grew as cell clusters under yeast growth conditions showed defects in filamentation under different hyphal growth conditions and were almost completely disabled for invasive filamentous growth. Under hyphal growth conditions only a fraction of *age3*Δ cells shows a wild-type-like polarization pattern of the actin cytoskeleton and lipid rafts. Moreover, *age3*Δ cells were highly susceptible to several unrelated toxic compounds including antifungal azole drugs. Irrespective of the *AGE3* genotype, C-terminal fusions of GFP to the drug efflux pumps Cdr1p and Mdr1p were predominantly localized in the plasma membrane. Moreover, the plasma membranes of wild-type and *age3*Δ mutant cells contained similar amounts of Cdr1p, Cdr2p and Mdr1p.

**Conclusions/Significance:**

The results indicate that the defect in sustaining filament elongation is probably caused by the failure of *age3*Δ cells to polarize the actin cytoskeleton and possibly of inefficient endocytosis. The high susceptibility of *age3*Δ cells to azoles is not caused by inefficient transport of efflux pumps to the cell membrane. A possible role of a vacuolar defect of *age3*Δ cells in drug susceptibility is proposed and discussed. In conclusion, our study shows that the ARF-GAP Age3p is required for hyphal growth which is an important virulence factor of *C. albicans* and essential for detoxification of azole drugs which are routinely used for antifungal therapy. Thus, it represents a promising antifungal drug target.

## Introduction

*Candida albicans* is one of the most prevalent human fungal pathogens. Depending on environmental conditions it is able to grow in several distinct cell forms, such as yeast cells, different pseudohyphal forms and true hyphae [1], [2]. Apart from other properties of *C. albicans* [3] hyphal development strongly contributes to its success as a pathogen [4].

Hyphal growth of *C. albicans* can be induced *in vitro* by varying growth conditions [5] and is controlled by a complex network of transcriptional activators and repressors [6], [7]. Recently, the group of David Kadosh and we independently identified a new central activator of hyphal development, *UME6*, that is required under all conditions of hyphal induction [8], [9]. Hyphae formation starts from a yeast-form cell (blastospore) by forming a germ tube which elongates until the first cell division. Before branching of the filament, further cell divisions in the growing filament take place only in the apical cell. Germ tube formation and hyphal elongation are the result of polarized growth [10]. The latter depends on the polarization of the actin cytoskeleton [11]–[14]. Several other hyphae-specific structures or properties depend on the polarized actin cytoskeleton. Among these, at the hyphal tip the Spitzenkörper (tip body), a hyphae-specific organelle closely beneath the polarisome, is present [15] and lipid rafts (sterol- and sphingolipid-enriched membrane domains) are highly concentrated at the tip [16].

Mucosal and systemic infections caused by *C. albicans* and other *Candida* species are treated by drugs belonging to several different chemical classes, e.g. azoles, polyenes and echinocandins [17]. However, antifungal therapy is often not successful and has become a serious problem due to the emergence of multidrug-resistant strains that result from extended use of antifungal drugs over the last decades [18]. Many *Candida* species including *C. albicans* have a high natural tolerance for antifungal drugs. Several highly potent drug efflux pumps that reside in the cytoplasmic membrane have different but overlapping substrate spectra to transport toxic compounds out of the cell [19]. There are two families of drug transporters. The ABC (ATP-binding cassette)-transporter family, which includes Cdr1p and Cdr2p, use the energy of ATP hydrolysis to extrude their substrates. The MFS (major facilitator superfamily) proteins (e.g. Mdr1p) use a drug/proton antiport system. Among other mechanisms, multidrug resistance of clinical *Candida* strains is often caused by higher expression of genes encoding drug efflux pumps [19]–[21]. Taken together, there is a high demand for the development of new antifungal drugs and the identification of potential drug targets.

The *Saccharomyces cerevisiae* gene *GCS1* [22], [23] encodes an ARF-GAP (ADP-ribosylation factor (ARF) GTPase-activating protein (GAP)) [24]. Several recent reviews discuss the various functions and properties of ARF proteins and ARF-GAPs in detail [25]–[27]. The GTP-bound form of ARF proteins is required for vesicle coat formation. Uncoating and formation of the naked transport vesicle is triggered by the GTPase activity of ARFs, which strictly depends on activation by ARF-GAPs. Gcs1p activates the intrinsic activity of Arf1p, Arf2p [24] and Arl1p (ARF-like protein 1) [28]. In *S. cerevisiae* for the products of four genes an ARF-GAP activity has been demonstrated [29]: *GCS1*, *GLO3*, *AGE1* and *AGE2*. The functions of these proteins in distinct intracellular vesicle routes partially overlap. The *C. albicans* genome carries homologs for each of these genes. *GCS1* appears to be the most important ARF-GAP in yeast because it shows synthetic lethality with other ARF-GAPs [29], is involved in several routes of intracellular vesicle traffic and has functions in both exocytosis and endocytosis [28], [30]–[32]. Colocalization studies revealed that Gcs1p is predominantly present in Golgi and endosomal compartments [33].

Apart from its ARF-GAP activity Gcs1p is also required for other processes in vesicle traffic, such as vesicle coat formation and vesicle docking with the target membrane by interaction with v-SNAREs [33], [34]. Furthermore, Gcs1p is required for maintenance of mitochondrial morphology [35], for formation of the prospore membrane in sporulation [36] and, like ARF-GAPs of other organisms, for the proper actin cytoskeletal organization by stimulating actin polymerization [27], [37].

In a systematic phenotypic study of *C. albicans* homologs of genes essentially required for ascospore formation in *S. cerevisiae*, we deleted the *C. albicans* homolog (orf19.3683) of *ScGCS1*. In consensus with Epp *et al.* [38], who have shown very recently that cells lacking this ORF are nearly avirulent in the murine model of disseminated infection and are killed by azole drugs instead of being growth inhibited, we call this *C. albicans* homolog “*AGE3*”, because the gene name “*GCS1*” has been used already for the *C. albicans* ortholog of the *S. cerevisiae* gene *GSH1* [39].

Here we show that *AGE3* complements several defects of the *S. cerevisiae gcs1*Δ strain. Thus, *AGE3* is indeed the functional ortholog of yeast *GCS1*. A clear delay of endocytosis in *age3*Δ cells is also shown. Mutant *age3*Δ cells have a severe defect in filament formation under several different hyphal growth conditions. We found that this is probably caused by defects in polarization of the actin cytoskeleton and lipid rafts of *age3*Δ cells. We also show that *age3*Δ cells are highly susceptible to several unrelated toxic compounds including azoles which are in use in antifungal therapy. Results of three experiments strongly indicate that the drug susceptibility of *age3*Δ cells is not the consequence of inefficient transport to the cell membrane or low activity of the drug efflux pumps Cdr1p, Cdr2p and Mdr1p.

## Materials and Methods

### Strains, media and growth conditions

The strains used in this study are listed in Table 1. For growing *S. cerevisiae* and *C. albicans* strains in the yeast-form, the cells were cultured at 30°C either in YPD (1% yeast extract, 2% bactopeptone and 2% dextrose) or in SD medium (0.17% yeast nitrogen base, 0.5% ammonium sulfate, 2% dextrose) supplemented with the appropriate amino acids for auxotrophic strains.

**Table 1:** *C. albicans* and *S. cerevisiae* strains used in this study.

Strain	Parent	Relevant genotype (otherwise see parent genotype)	Source
***C. albicans*** ** strains**			
SC5314		Wild-type clinical isolate	[80]
SN87	SC5314	*leu2*Δ/*leu2*Δ *his1*Δ/*his1*Δ *URA3/ura3*Δ *IRO1/iro1*Δ	[47]
UZ22	SN87	*age3*Δ::*CmLEU2/AGE3 his1*Δ/*his1*Δ	This study
UZ45	UZ22	*age3*Δ::*CdHIS1/age3*Δ::*CmLEU2*	This study
UZ55	UZ45	*age3*Δ*/age3*Δ::*AGE3* (*P_ACT1_-CaSAT1*)*	This study
TL19	SC5314	*AGE3*/*AGE3 CDR1*/*CDR1 RPS1*/*rps1*::P_tet_-*CDR1*-GFP (P*_ACT1_*-HygB^R^)*	This study
TL20	UZ45	*age3*Δ*/age3*Δ *CDR1*/*CDR1 RPS1*/*rps1*::P_tet_-*CDR1*-GFP (P*_ACT1_*-HygB^R^)*	This study
TL21	SC5314	*AGE3*/*AGE3 MDR1*/*MDR1*-GFP (P_tet_-*MDR1* P*_ACT1_*-HygB^R^)*	This study
TL22	UZ45	*age3*Δ*/age3*Δ *MDR1*/*MDR1*-GFP (P_tet_-*MDR1* P*_ACT1_*-HygB^R^)*	This study
***S. cerevisiae*** ** strains**			
BY4743	S288C	Mat a/α *his3*Δ1/*his3*Δ1 *leu2*Δ0/*leu2*Δ0	[57]
ydl226c+/−	BY4743	*gcs1*Δ::kanMX4/*GCS1*	[81]
ydl226c−/−	BY4743	*gcs1*Δ::kanMX4/*gcs1*Δ::kanMX4	[78]
UZ177	ydl226c−/−	*gcs1*Δ::kanMX4/*gcs1*Δ::kanMX4 (centromeric plasmid pCaAge3-2: *URA3 P_Tet_-CaAGE3*)	This study

*Gene fusions in parentheses denote integration of a complete plasmid carrying these fusions into the locus indicated.

For sporulation of diploid *S. cerevisiae* strains, cells were removed from YPD agar after fresh growth in patches of 1×1 cm for 16 hours at 30°C, resuspended in 1% potasium acetate at a concentration of 1×10^8^ cells per ml and 100 µl of the suspension were spotted onto 1% pottasium acetate agar containing 50 µg/ml doxycycline (and for control, without doxycycline). After five days of spore formation at 28°C, the cells were removed from the plates, resuspended and the sporulation efficiency determined after microscopic quantification.

For filamentous growth of *C. albicans* in liquid media the cells were incubated at 37°C in either of the following media: YPD +10% bovine serum with or without buffering with 20 mM potassium phosphate to pH 7.5, 2.5 mM *N*-acetylglucosamine (GlcNAc) in 0.335% yeast nitrogen base with ammonium sulfate, RPMI-1640 (GIBCO-BRL) or Spider medium [40]. Agar plates (2% agar) for filamentous growth were prepared using Spider medium. For filamentous growth under embedded conditions at 37°C YPS agar (1% yeast extract, 2% bactopeptone, 2% sucrose, 1% agar) was prepared and the cells embedded as described [41].

### Plasmid constructions

Oligonucleotide primers used for PCR amplification and all plasmids used and constructed are listed in Tables S1 and S2, respectively.

Construction of pCaAge3-Sat2 used for reintegration of the *AGE3* gene into the homozygous *age3*Δ strain was done as follows. First, a gene cassette which is composed of the *SAT1* gene (conferring nourseothricin resistance) located between the *ACT1* promoter and the *ADH1* terminator sequence was PCR-amplified from plasmid pSDS4 (Bito A., unpublished). The latter carries modified fragments from pSFS1A [42] and the cassette was amplified using the oligonucleotides CaAct1-Spe-Nar und CaAdh1-Aat followed by digestion with *Nar*I and *Aat*II. This cassette was cloned into YCplac33 [43], resulting in plasmid pCaAct-Sat1. The *AGE3* gene including 100 bp upstream of the ORF was PCR-amplified with oligonucleotides CaAge3-9 and CA0423-2. The PCR product was digested with *Sal*I and *Sma*I and inserted into pCaAct-Sat1 resulting in plasmid pCaAge3-Sat2. The nucleotide sequence of the *AGE3* gene was verified.

The plasmid pCaAge3-2 used for complementation of the yeast *gcs1*Δ strain by *CaAGE3* was constructed by amplification of the *AGE3* ORF from *C. albicans* genomic DNA using the primers CaAge3-7 and CaAge3-8, digestion of the PCR product by *Bam*HI and insertion into plasmid pCM252-U. The latter was derived from pCM252 [44] by replacement of the resident *ScTRP1* gene (*Sca*I fragment) by *ScURA3* which had been isolated from YCplac33 [43] by partial digestion with *Sca*I.

Plasmid pTet-GFP which carries a *C. albicans* codon-optimized hygromycin B resistance gene (Wong *et al.*, poster 89B presented at the 7th ASM conference on Candida and Candidiasis, Austin, Texas, USA) was the starting plasmid for generating C-terminal GFP fusions. It was constructed in five steps and included fragments of pAU22 [45] and CIp10 [46]. A detailed description of the cloning steps and the plasmid maps are shown in Fig. S2. The plasmids pCdr1-GFP and pMdr1-GFP encoding the respective fusion proteins were constructed by insertion of PCR products amplified by the primer pairs CaCdr1-Sal/CaCdr1-Ngo and CaMdr1-Eco/CaMdr1-Ngo, respectively. The PCR products and the vector were digested with *Ngo*MIV and *Eco*RI (*MDR1* ORF) or *Ngo*MIV and *Sal*I (*CDR1* ORF) and ligated.

### *C. albicans* strain constructions

The strains used are listed in Table 1.

The heterozygous and homozygous *age3* deletion strains, UZ22 and UZ45, were constructed as described by Noble and Johnson [47]. To this end, the regions (about 300 bp) immediately upstream and downstream of the *AGE3* open reading frame, and the marker gene cassettes *CdHIS1* and *CmLEU2* were amplified either from the *Candida* genome or from the plasmids pSN52 and pSN40 [47], respectively. The PCR primers used were CaAge3-d1 and CaAge3-d3, CaAge3-d4 and CaAge3-d6, and CaUniv-2 and CaUniv-5 for the region immediately upstream and downstream of the *AGE3* ORF and the auxotrophic marker genes, respectively (Table S1). The gene deletion cassette was generated by fusion PCR and integrated into the genome of strain SN87 [47]. The *AGE3*-reconstituted strain UZ55 was generated by integration of plasmid pCaAge3-Sat2 (Table S2), which had been linearized before by *Bsp*1407I digestion, at the native *AGE3* promoter of the *age3*Δ strain, thereby reconstituting one wild-type *AGE3* gene copy. After transformation, recombinant clones were selected on YPD agar containing 200 mg/L nourseothricin. For both the homozygous *age3* deletion strains and the reconstituted strains the expected structural genomic alterations were confirmed by both PCR (not shown) and Southern blots (Fig. S1).

To construct strains that encode C-terminal fusions of GFP [48] to Cdr1p and Mdr1p, respectively, the plasmid pCdr1-GFP was linearized with the restriction enzyme *Age*I and inserted into one allele of the *C. albicans RPS1* gene, whereas pMdr1-GFP was linearized with *Kpn*I and inserted into the *MDR1* ORF. After transformation of the wild-type strain SC5314 and the *age3*Δ strain with pCdr1-GFP or pMdr1-GFP, the transformants were selected on agar plates containing 200 µg/ml (for the *age3*Δ strain) or 1000 µg/ml hygromycin B (wild-type strain). Plasmid integration at the expected loci was confirmed by PCR. As the consequence of the different integration sites the expression of the *CDR1*-GFP cassette is controlled by the Tet promoter [49] whereas the *MDR1*-GFP cassette is controlled by the *MDR1* promoter. Thus, chimeric gene expression of cells was induced by addition of 50 µg/ml doxycycline (Cdr1p-GFP) or 25 µg/ml benomyl (Mdr1p-GFP) for four hours.

### RNA isolation, Northern blotting and quantitative reverse transcriptase PCR (qRT-PCR)

Total RNA was isolated from cells grown to logarithmic phase in YPD or induced for filamentous growth in YPD+serum by using the RNeasy® Mini Kit from Qiagen. Northern blot analysis was done as described elsewhere [9]. For synthesis of the *AGE3*-specific probe, a PCR product was amplified by using the oligonucleotide primers CaAge3-4 and CaAge3-5 (Table S1). The antisense strand was labelled by incorporation of [α-^32^P]-dATP in a single-stranded PCR reaction. After hybridization, transcripts were made visible by phosphoimaging (Fujifilm BAS-1800 II phosphoimager). As a control for RNA quantity and transfer efficiency rRNA was stained on the blot with methylene blue.

Quantitative *AGE3* gene expression was determined by qRT-PCR analysis. After degrading residual DNA from the RNA samples by DNaseI cDNA synthesis was performed using the GoScript™ Reverse Transcription System (Promega Corp.). Gene-specific fragments of *ACT1* (control for constitutive expression) and *AGE3* were amplified by real-time PCR (primer pairs CaAct1-10/CaAct1-11 and CaAge3-9A/CaAge3-10, respectively) in a Corbett Rotor-Gene RG-3000 machine using the GoTaq® qPCR Master Mix (Promega Corp.). The relative *AGE3* expression levels of different strains were calculated by the ΔΔCt method (normalization to *ACT1* expression). For each cDNA sample real-time PCR was done in duplicate and the experiments were repeated once on a different day for reproducibility.

### Plasma membrane isolation and Western blotting

The plasma membranes of *Candida* yeast cells grown in YPD to exponential phase (OD_600_ = 1.0) were purified as described by Panaretou and Piper [50]. For induction of *CDR1* and *CDR2* expression 10 µg/ml β-estradiol [51], and for *MDR1* induction 25 µg/ml benomyl had been added to the medium for one hour before harvesting the cells. After separation of membrane proteins (10 µg) by SDS-PAGE (10% polyacrylamide gels) and blotting onto nitrocellulose membranes, the blots were incubated with polyclonal antibodies raised against Cdr1p, Cdr2p or Mdr1p (kindly provided by D. Sanglard) [52]. After incubation with secondary antibodies (goat anti rabbit) conjugated with alkaline phosphatase (AP) the drug transporters were visualized by staining the blots with the AP substrate 5-bromo-4-chloro-3-indolyl phosphate (BCIP) and nitro blue tetrazolium (NBT).

### Staining procedures, fluorescence microscopy and rhodamine 6G influx/efflux

Endocytosis under yeast growth conditions was investigated by staining of endocytotic vesicles with FM4-64 [53] as follows. Cells were grown in YPD to an OD_600_ of 0.5, harvested by centrifugation and resuspended in 300 µl YPD containing 2.5 µl FM4-64 stock solution (stock 1.64 mM). The samples were incubated at 0°C for 40 minutes and then washed in 1 ml YPD. Then the cells were resuspended in 200 µl YPD to start of endocytosis and incubated for different time periods at 30°C. Samples were taken and washed in 1× PBS before microscopic analysis.

Hyphal structures were stained according to procedures formerly published. Lipid rafts were stained with filipin [16] using a concentration of 16.6 µg/ml. F-actin was stained as described [54] with the modification that instead of rhodamine-conjugated phalloidin Alexafluor-488-conjugated phalloidin was used.

The structures were inspected with a Leica TCS SP5 confocal microcroscope using the appropriate excitation and emission wave lengths.

The steady-state level of intracellular rhodamine 6G was determined using the protocol of Maesaki *et al.* [55]. The influx and efflux of rhodamine 6G was measured as described [56].

### Testing susceptibility to metabolic inhibitors and zymolyase

To test strains for sensitivity to metabolic inhibitors, strains were initially grown for 16 hours in YPD medium. Then the cell suspensions were diluted in YPD to an OD_600_ of 0.05 and the cells grown at 30°C for six hours. The cell number per ml was determined with a hemacytometer and serially diluted in cold H_2_O. Each five µl suspension containing about 20, 200, 2.000 or 20.000 cells were spotted onto YPD agar containing certain toxic compounds. For each compound three concentrations were initially tested for optimal results. After two or three days of growth at 30°C the agar plates were photographed. Growth susceptibility of *S. cerevisiae* strains to 80 mM NaF in YPD was tested by inoculating in medium with and without 50 µg/ml doxycycline and incubation for 46 hours at 30°C. The OD_600_ was measured at certain time points.

Sensitivity to zymolyase™ (Seikagaku Biobusiness Corp., Japan) was tested as follows. The strains (wild type, *age3*Δ and reintegrant) were grown in YPD at 30°C to exponential phase (OD_600_ about 0.5). After washing with water, the cells were resuspended in SCE+DTT (1 M Sorbitol, 10 mM Na-Citrat (pH 7.5), 20 mM EDTA, 20 mM DTT) for 30 minutes at 23°C. The cells were washed in SCE and an amount of 2.5×10^7^ cells per ml SCE including zymolyase at a concentration of 5, 10 or 20 µg/ml was incubated at 37°C for 30 minutes. One volume of 2% SDS was added and the OD_600_ measured. The percentage of viable cells was determined according to the following equation: % viable cells  = 100× (OD_600_ with zymolyase/OD_600_ of a control sample without zymolyase).

## Results

### Deletion of *AGE3* from the *C. albicans* genome and gene expression

To study the biological role of *AGE3* in *C. albicans* we made heterozygous and homozygous strains, UZ22 and UZ45 (Table 1), respectively, that lack one or both alleles of *AGE3*. In addition, as a control for phenotypic analyses an *AGE3*-reconstituted (“reintegrant”) strain, UZ55, was generated. In this strain, one copy of the wild-type *AGE3* gene had been reintegrated into the *age3*Δ strain at the native genomic locus.

**Figure 1:**
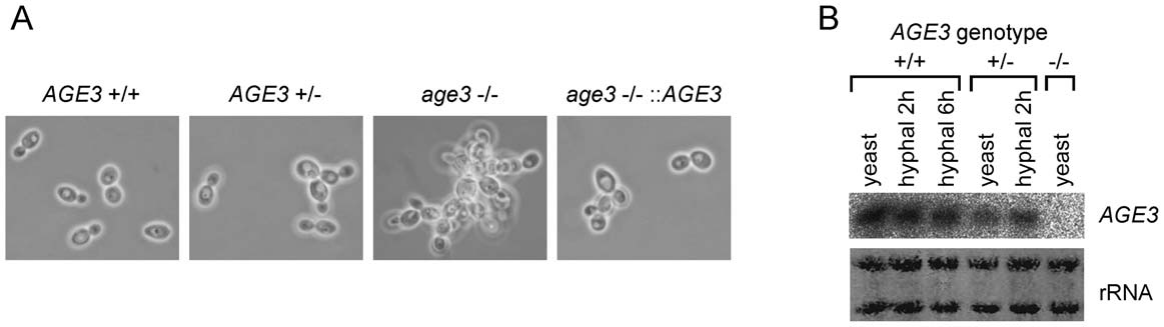
Morphology of the *age3*Δ/Δ strain under yeast growth conditions and *AGE3* gene expression. (A) The strains SN87 (*AGE3*+/+), UZ22 (*AGE3*+/Δ), UZ45 (*age3*Δ/Δ) and UZ55 (*age3*Δ/Δ::*AGE3*) were grown in SD medium at 30°C to an OD_600_ of 0.5. Samples were inspected under the microscope (phase contrast optics). Note that the homozygous *age3* mutant strain forms aggregates of yeast cells. The heterozygous and the reintegrant strain show intermediate phenotype. (B) Northern blotting of RNA isolated under yeast and hyphal growth conditions. The wild-type strain, the heterozygous and the homozygous *age3* mutant strains were grown either to exponential phase in YPD or induced for hyphal growth (two and six hours) in YPD +10% bovine serum. Each 4 µg total RNA were blotted and hybridized with a ^32^P-labeled *AGE3*-specific probe. Methylene blue-stained rRNA on the blot is shown as a control for blotting efficiency.

Both in YPD and SC medium *age3*Δ yeast cells formed aggregates (Fig. 1A) which disintegrate when the coverslip and the cells in suspension beneath are gently pressed to the slide. This indicates that *age3* mutant cells possibly adhere to each other or have some defects in cell separation after division.

Gene expression analysis by Northern blotting (Fig. 1B) and qRT-PCR analysis (Table 2) revealed that the gene is expressed constitutively at similar high levels under yeast and hyphal growth conditions. Under these conditions both the heterozygous and the reintegrant strains showed a lower gene expression compared to the wild type. This is probably the reason for the intermediate phenotype (between wild-type and *age3* mutant) shown by the heterozygote and the reintegrant strains in most of the experiments described in this study. The different *AGE3* expression levels of the reintegrant strain and the heterozygous strain could be caused possibly by expression of the gene from the promoters of different alleles (was not investigated), because the promoter regions of the two *AGE3* alleles differ from each other by several nucleotide insertions and deletions.

**Table 2:** *AGE3* transcript levels in heterozygous and homozygous *age3* mutant cells.

Strain	*AGE3* genotype	Growth condition^1^	Relative *AGE3* transcript level^2^
SN87	+/+	Yeast	1.00
		Hyphae 2 hours	1.01
		Hyphae 6 hours	0.93
UZ22	+/−	Yeast	0.45
		Hyphae 2 hours	0.44
UZ45	−/−	Yeast	0.00
UZ55	−/− (+)	Yeast	0.69
		Hyphae 2 hours	0.63

1 The cells were grown either in YPD at 30°C to exponential phase (yeast) or induced for hyphal growth (hyphae) in YPD + bovine serum at 37°C for two or six hours.

2 The transcript levels were determined by qRT-PCR and are given as mean levels of two parallel reactions compared to the *AGE3* expression level of wild-type cells grown under yeast growth conditions.

### *AGE3* is the functional ortholog of *S. cerevisiae GCS1*

**Figure 2:**
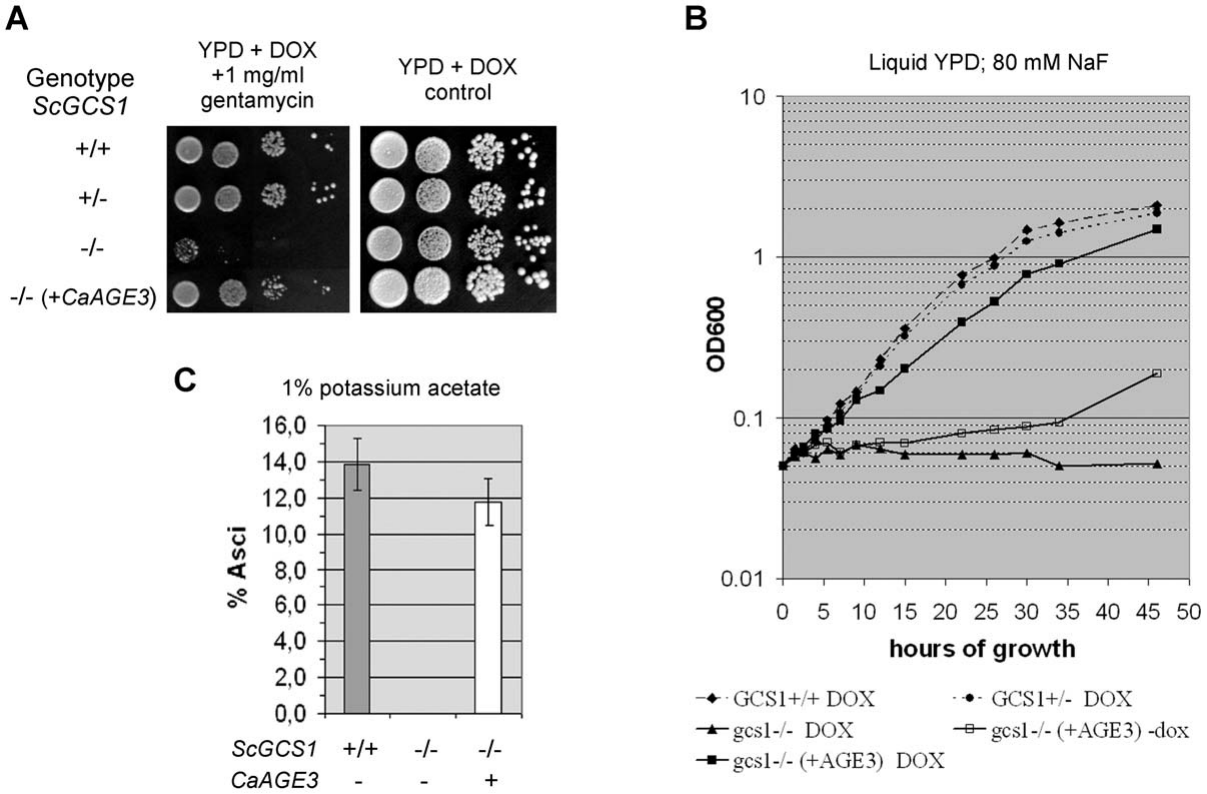
*CaAGE3* complements defects of the yeast *gcs1*Δ mutant. (A) The diploid *S. cerevisiae* strains BY4743 (wild type), ydl226c+/− (heterozygous for *GCS1*), ydl226c−/− (homozygous *gcs1*Δ) and UZ177 (homozygous *gcs1*Δ transformed with the centromeric plasmid pCaAge3-2 which carries the *CaAGE3* ORF under control of the doxycycline-inducible Tet promoter) were grown for 48 hours on agar plates containing 50 µg/ml doxycycline (DOX) in the presence of gentamycin (A). The number of cells spotted onto the agar was about 20,000, 2,000, 200 or 20 (from left to right). (B) Exponentially pregrown cells (strains as in A) were inoculated into 80 mM sodium fluoride in YPD +50 µg/ml DOX and incubated at 30°C for 42 hours. Samples were taken after different time points and the OD_600_ measured. For control, each strain was also grown without DOX. Except for strain UZ177 the growth rate was independent of the presence of DOX (not shown). However, *AGE3* induction in strain UZ177 by DOX results in complementation of the growth deficiency of *gcs1*Δ cells. This experiment was repeated and very similar growth behaviours were observed. (C) The wild-type, the homozygous *gcs1*Δ strain and UZ177 were induced for sporulation on agar plates containing 1% potassium acetate and 50 µg/ml DOX. The percentage of asci formation was determined after five days incubation at 30°C. This experiment was done in triplicate. Expression of *CaAGE3* complements the complete inability of the *gcs1*Δ/Δ strain to form asci. Note that in general the sporulation efficiency of strains derived from BY4743 is low compared to other wild-type strains.

The open reading frames (ORFs) of *C. albicans AGE3* and *S. cerevisiae GCS1* encode for proteins of 379 and 352 amino acids length, respectively. The protein sequences are 46.7% identical and 54.3% similar (E-value: 1.2e-89). The N-terminal ARF-GAP domains show the highest homology (83.6% identity, 91.0% similarity). To confirm that *AGE3* is indeed the functional ortholog of *GCS1* we investigated whether *AGE3* is able to complement some known defects of the yeast *gcs1*Δ strain. To this end, the *AGE3* ORF was cloned behind the doxycycline-dependent Tet promoter [44] on the centromeric plasmid pCM252-U. The resulting plasmid pCaAge3-2 was transformed into the homozygous diploid yeast *gcs1*Δ strain ydl226c−/− from the EUROSCARF collection [57]. The resulting strain was called UZ177. We then compared the ability of UZ177, the *gcs1*Δ strain and the wild-type strain for gentamycin susceptibility and sporulation efficiency, which depend on Golgi and vacuole functions [58] and proper formation of the prospore membrane [36]. Furthermore, the growth in the presence of sodium fluoride which inhibits glycolysis by binding to enolase [59] was compared. In each case the wild-type phenotype was nearly fully restored in the mutant strain when the *C. albicans AGE3* ORF was expressed (Fig. 2). This suggests that *C. albicans AGE3* is indeed the functional ortholog of *S. cerevisiae GCS1* and probably functions as an ARF-GAP. In parallel we found that doxycycline did not have any influence on growth in liquid media on any *S. cerevisiae* strain used in this study (not shown).

### *age3* mutant cells show a strong delay in endocytosis

**Figure 3:**
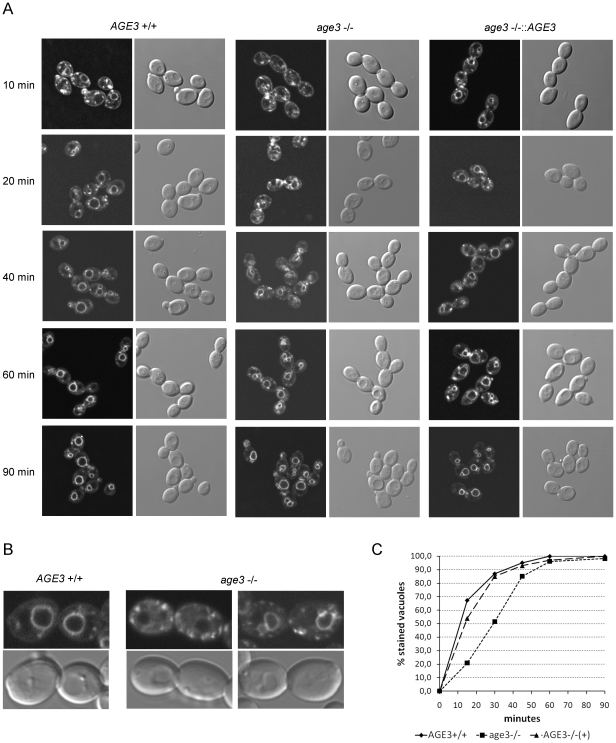
Cells lacking *AGE3* show a clear delay in endocytosis. (A) Cells of the strains SN87 (*AGE3*+/+), UZ45 (*age3*Δ/Δ) and UZ55 (*age3*Δ/Δ::*AGE3*) were grown in YPD to exponential phase. After staining the cytoplasmic membrane with the lipophilic fluorescent dye FM4-64 at 0°C for 40 minutes, the excess dye was removed by washing. Then the cells were released for growth in YPD at 30°C to allow endocytosis to occur. Samples were taken at the indicated time points. Staining of endocytic vesicles and the vacuole was visualized by confocal fluorescence microscopy. DIC images are shown for comparison. (B) One and two cell pairs of the wild-type and the *age3*Δ/Δ mutant strains, respectively, which were harvested 40 minutes after release of endocytosis are shown in higher magnification. The mutant cells show stronger background staining of the cytoplasm and more bright spots compared to the wild-type cells. The vauolar membranes of the left mutant cells still have not taken up FM4-64. (C) Quantification of FM4-64 uptake into vacuolar membranes in cells of the strains mentioned above. Images of a similar experiment as described under (A) were analysed and the percentage of cells (about 100–150 in total for each strain and time point) with clearly stained vacuolar membranes determined. A similar experiment performed on another day with slightly different time points showed a very similar FM4-64 uptake kinetics.

To confirm that *age3*Δ cells have defects in intracellular vesicle traffic, we studied their endocytotic efficiency after staining the cytoplasmic membrane with the lipophilic fluorescent dye FM4-64. Although FM4-64 was internalized efficiently, many *age3*Δ cells showed bright fluorescent spots in the cytoplasm or irregular structures (even at later time points) which was observed less often in cells of the wild-type and reintegrant strains (Fig. 3A and 3B). Moreover, the *age3* mutant cells had a clear delay of endocytosis compared to wild-type cells. In contrast to mutant cells the vacuolar membranes of many wild-type and reintegrant cells were stained weakly even ten minutes after release of endocytosis (temperature shift from 0 to 30°C after removing excess FM4-64) (Fig. 3A). Compared to wild-type cells it took about 20 minutes longer (this corresponds to about 1/3 of the doubling time) until 50% of the mutant cells had taken up FM4-64 into the vacuolar membrane (Fig. 3C). Although all mutant cells showed staining of the vacuolar membrane after one hour, there were still several bright spots observed in the cytoplasm and close to the cell membrane of many mutant cells (Fig. 3B). This and the generally higher background staining of cytoplasm indicates that mutant cells also endocytose quantitatively less efficiently than wild-type cells.

### The *age3*Δ strain has severe defects in hyphal and invasive growth under several distinct conditions

**Figure 4:**
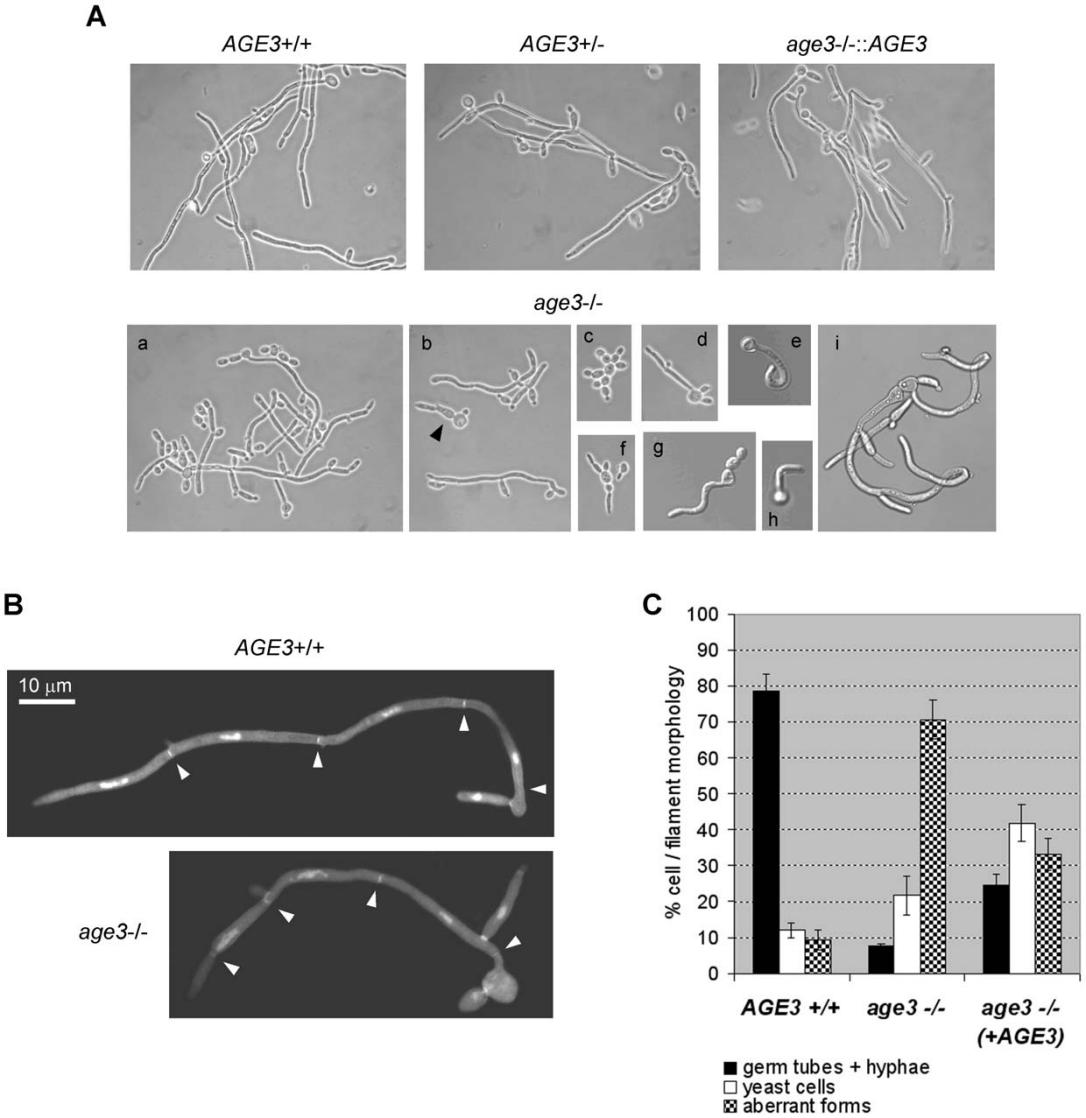
Growth forms of the *age3*Δ/Δ strain after hyphal induction in YPD +10% bovine serum (A and B) or in GlcNAc medium (C). (A) The strains SN87 (*AGE3*+/+), UZ22 (*AGE3*+/Δ), UZ45 (*age3*Δ/Δ) and UZ55 (*age3*Δ/Δ::*AGE3*) were induced for hyphal growth in YPD +10% bovine serum at 37°C. After five hours of growth, samples were inspected under the microscope (most pictures in phase contrast and some in Nomarsky optics). Note that the homozygous *age3* mutant strain forms several distinct forms of filaments. Some of them are not true hyphal filaments. Most strikingly, a fraction of germ tubes and filaments show an untypically curved or spiral shape (images e, g, h and i). Some other filaments show constrictions between cells which are typical for pseudohyphae (i). There are also cell clusters seen which are composed of yeast-form cells (c) and others which show only poor polarized growth (d, f and b arrow head). (B) Each one true hyphal filament of the wild-type and the *age3* mutant strains grown under the same conditions as in (A) were stained with Calcofluor white and DAPI to visualize septa (arrow heads) and nuclei, respectively. Compared to wild-type filaments, *age3* mutant filaments are composed of shorter hyphal cells (except for the first cell following the blastospore in many cases). (C) The strains SN87 (*AGE3*+/+), UZ45 (*age3*Δ/Δ) and UZ55 (*age3*Δ/Δ::*AGE3*) were induced for hyphal growth in GlcNAc-containing medium. After three hours of growth at 37°C for each clone about 200 cells and filaments were classified as wild-type-like germ tubes and hyphae, yeast cells and aberrant forms of single cells, germ tubes or filaments (curved shape, short pseudohyphal elements, cells with thick germ tubes; as shown in (A)). The experiment was done in triplicate. The mean percentage of these morphologies and standard deviation are shown.

We then studied filamentation of *age3*Δ cells under several distinct growth conditions, both in liquid media, on agar surfaces and when embedded in agar. Depending on the growth conditions, the mutant strain showed more or less severe defects in formation of long filaments in liquid media. True hyphae formation of the *age3*Δ strain was most efficient in YPD containing 10% bovine serum and germ tube formation occurred as efficiently as in wild-type background (albeit with a short delay and a reduced germ tube length compared to wild-type cells). However, during prolonged growth only 54.3% (standard deviation (SD)  = 4.6; about 400 cells of three cultures each were determined) of the mutant cells further developed into filaments of normal true hyphae appearance, whereas 91.3% (SD = 1.4) of wild-type cells and 75.6% (SD = 3.9) of the heterozygous and 79.6 (SD = 2.4) of the reintegrated cells showed normal appearance. The rest of the mutant cells were single yeast cells, cells of aberrant shape and also single cells with bent shape of the germ tube were observed (Fig. 4A). Many filaments were shorter, sometimes of undulating or helical shape and some appeared swollen at the end of the apical cell. Filaments and cells grown in serum-containing medium form huge aggregates. Fixation in ethanol and subsequent separation of the filaments by digesting proteins with pepsin resulted in osmotic shrinkage of filaments and made it very difficult to distinguish filaments of different diameter and shape. Therefore we were not able to determine the fractions of different aberant cell and filament forms reliably. Filaments of “normal” appearance (without fixation) were composed of hyphal cells with a mean length of about 73% of wild-type cells (Fig. 4B). The mean lengths were 27.4 µm (±3.44) for wild-type hyphal cells and 20.1 µm (±1.69) for mutant cells. However, the first hyphal cell of mutant filaments was often as long as in wild-type filaments. A larger difference of hyphae formation between *age3*Δ and wild-type cells was observed in medium containing *N*-acetylglucosamine (GlcNAc) (Fig. 4C), which strongly induces hyphal growth of wild-type cells. Whereas around 80% of the wild-type cells germinated and formed true hyphae of up to three cells three hours after hyphal induction, only a fraction of about 8% of the *age3*Δ cells germinated and the few filaments formed were composed of no more than two cells. Many cells had an aberrant shape. After five hours nearly 100% of the wild-type blastospores germinated or the filaments had been elongated further, whereas most of the mutant “filaments” were not able to elongate or divide further (not shown). Also in Spider medium the *age3*Δ cells formed very few short filaments (not shown). Most mutant cells were of aberrant and often bent shape. The heterozygous and the reintegrant strains showed an intermediate phenotype in all media.

In conclusion, *age3*Δ cells are strongly compromised in maintaining polarized filamentous growth after germ tube formation in liquid media (the severity of these defects depending on the medium).

**Figure 5:**
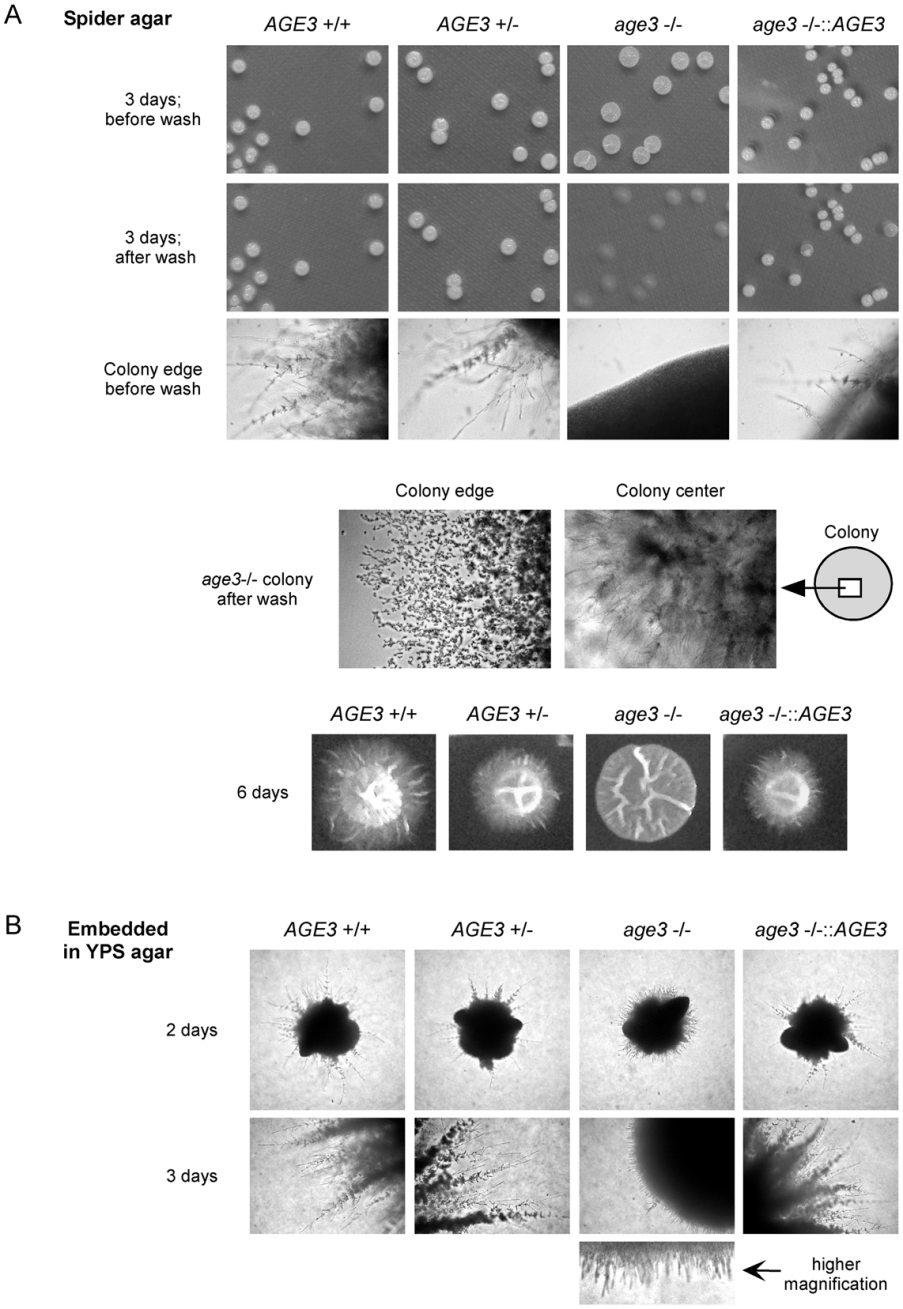
Defective filamentous and invasive growth of the *age3*Δ/Δ strain on/in solid media. Note: All images of each row are shown in the same magnification. (A) The strains SN87 (*AGE3*+/+), UZ22 (*AGE3*+/Δ), UZ45 (*age3*Δ/Δ) and UZ55 (*age3*Δ/Δ::*AGE3*) were induced for filamentous growth on Spider agar at 37°C for three and six days (two sets of agar plates). The colonies were photographed and the colony edges inspected under the microscope. To try to remove the colonies after three days of growth one set of agar plates was washed with water. Only the homozygous *age3* mutant colonies could be removed easily. At the edges of the mutant colonies after wash single yeast cells and very short filaments are still attached to the agar. However, weak invasive growth into the agar by *age3* mutant filaments is only visible in the colony center. After six days, filamentous growth is seen macroscopically for the wild-type, the heterozygous and the reintegrant strain. The mutant strain forms flat colonies without visible filaments (as also seen after three days). (B) The strains mentioned under (A) were induced for filamentous growth by embedding in YPS agar and incubation for three days at 37°C. The homozygous *age3* mutant colonies formed after two days of growth show a high rate of filamentation. However, the filaments were unable to develop further (three days) into long filaments which could invade the agar as filaments of the control strains do.

We also investigated filamentous growth of the *age3* mutant when grown on Spider agar and when embedded in YPS agar (Fig. 5). Compared with liquid media, the mutant strain showed an even stronger filamentation defect on/in the agar media. Although being able to form long filaments in the center of the colonies grown on Spider agar, the colonies were easily washed from the agar surface (Fig. 5A). Moreover, the *age3*Δ colonies were very flat and larger compared to colonies formed by other strains. Under embedded conditions the *age3*Δ strain formed very short filaments instead of long and branched filaments observed with the wild-type strain (Fig. 5B). From these results we conclude that *AGE3* is essential for invasive filamentous growth in *C. albicans*.

### Filamentation deficiency of *age3*Δ cells correlates with improper organization of the actin cytoskeleton and the absence of lipid raft polarization at the hyphal tip

We next investigated whether *age3*Δ cells induced for hyphal growth show polarization of the actin cytoskeleton and lipid rafts. As in other forms of polarized growth of fungi the development of true hyphae depends on the polarization of actin cables, which is established by regulatory and structural cytoskeletal proteins and by cortical patches of actin [11]–[14], [54]. Actin was stained with Alexafluor-488 phalloidin. The high enrichment of lipid rafts at the hyphal tip [16] were stained with the polyene filipin, which binds to ergosterol [60].

**Figure 6:**
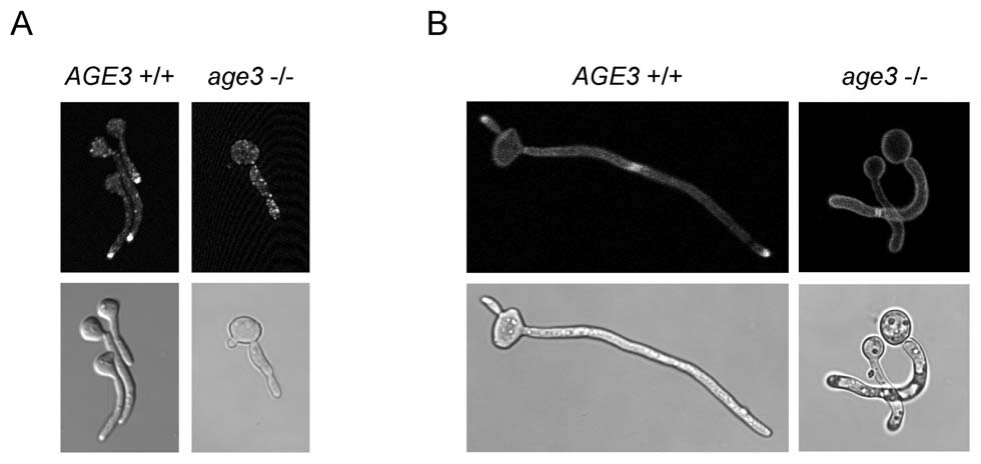
Absence of polarization of lipid rafts and actin patches in *age3*Δ cells. The strains SN87 (*AGE3*+/+) and UZ45 (*age3*Δ/Δ) were induced for hyphal growth in GlcNAc medium at 37°C. After three hours samples were taken and the cell structures shown were stained and visualized under the confocal microscope. For control, pictures made with phase contrast or DIC optics are shown below the fluorescence picture. (A) At the tips of germ tubes of wild-type cells the actin patches, which were stained with Alexafluor 488-conjugated phalloidin, are highly concentrated. Most mutant cells do not show this polarization of the actin cytoskeleton. (B) Lipid rafts were stained with filipin. As expected, lipid rafts are concentrated at the hyphal tips and at septa in the wild type. Most mutant germ tubes (note that some have constrictions at the neck) and filaments show a uniform staining of the cytoplasmic membrane. Note that the figure shows only typical structures found in cultures of wild-type and mutant cells. However, a low fraction of mutant cells and filaments (see Fig. 4C) showed a wild type-like staining patterns. Also for the reintegrant strain (not shown) both wild-type and mutant staining patterns were observed.

To study these structures, we induced mutant and wild-type cells for hyphal growth in GlcNAc-containing medium, because under these conditions we observed the mutant's strongest defect in hyphal growth. The wild-type strain showed the expected concentration of actin patches at the tips of germ tubes and hyphae (Fig. 6A). However, for most of the germ tubes (or germ tube-like structures) and short filaments formed by *age3*Δ blastospores, actin patches were scattered along the cell bodies and a polarization pattern was rarely observed. A similar difference was observed for the distribution of lipid rafts (Fig. 6B). Whereas wild-type hyphae showed the typical strong staining of hyphal tips and septa with filipin, ergosterol and lipid rafts were uniformly distributed in the cytoplasmic membrane of many *age3* mutant filaments and germ tubes. As mentioned, *age3*Δ cells and filaments show a certain variability in shape. Filaments which are morphologically similar to the wild-type hyphae showed nearly normal staining patterns of actin patches and lipid rafts (not shown). The reintegrant strain was observed in parallel and showed both wild-type and mutant staining patterns (not shown).

### Cells lacking *AGE3* are highly susceptible to several unrelated toxic compounds

**Figure 7:**
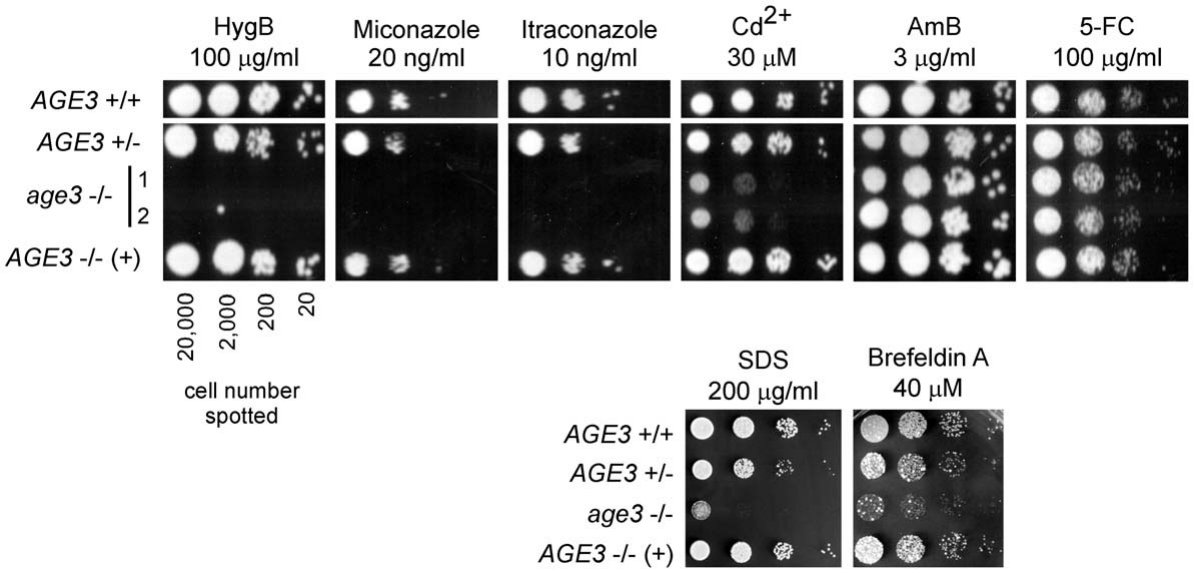
The *age3*Δ mutant strain is susceptible to several unrelated metabolic inhibitors. The strains SN87 (*AGE3*+/+), UZ22 (*AGE3*+/Δ), UZ45 (*age3*Δ/Δ) and UZ55 (*age3*Δ/Δ::*AGE3*) were grown in YPD medium at 30°C to an OD_600_ of 0.5. After washing, the cells were spotted in serial dilutions (cell number per spot is given) onto agar plates containing distinct concentrations of the toxic compounds mentioned and grown for two or three days at 30°C. The homozygous *age3* mutant cells are susceptible to the azole drugs miconazole and itraconazole, hygromycin B (HygB), cadmium ions, SDS and brefeldin A, but not to the antifungal drugs amphotericin B (AmB) and 5-fluorocytosine (5-FC). Note, that as observed for other phenotypes, the heterozygous and the reintegrant strain show an intermediate susceptibility between the wild-type and the homozygous mutant strains. In the experiments shown in the upper panel two independent clones (1 and 2) of the construction of the homozygous *age3*Δ strain were spotted. Since these clones behaved identical, only clone 1 was used in later experiments (lower panel).

To identify other cellular functions of the Age3 protein in *C. albicans* we determined the growth behaviour of the mutant strain in the presence of several distinct metabolic inhibitors. As shown in Fig. 7, the *age3*Δ strain showed high susceptibility to hygromycin B, the azole drugs miconazole and itraconazole, SDS, Cd^2+^ ions and brefeldin A. In the presence of 5-fluorocytosine and amphotericin B, which are both used in antifungal therapy in combination with or without azoles [19], we did not observe any influence on growth in the absence of *AGE3*. The sensitivity of mutant cells to brefeldin A, which is an inhibitor of ARF-GEFs (ARF guanine nucleotide exchange factors; these are required for restoring the potentially active ARF-GTP complex) [61], confirms a role of the Age3 protein in the process of vesicle uncoating. The toxic compounds identified have different chemical structures and are involved in inhibition of distinct cellular processes and structures, such as protein glycosylation, cell wall and protein synthesis (hygromycin B), ergosterol biosynthesis and lipid composition of the plasma membrane (azoles), as well as other membrane or cell wall defects (SDS), and heavy metal toxicity (cadmium ions). Since yeast Gcs1p, and probably *C. albicans* Age3p as well, function as ARF-GAPs and are required for efficient vesicle traffic, the pleiotropic susceptibility spectrum may be caused mainly by improper or defective intracellular vesicle and protein transport to different organelles and membranes of most *age3*Δ cells. In particular, the high sensitivity of *age3*Δ cells to azoles, hygromycin B and cadmium may be caused by inefficient transport of drug efflux pumps to the plasma membrane. As a consequence *age3* mutant cells could have a much lower capacity to remove these compounds from the cell.

### The drug efflux pumps Cdr1p, Cdr2p and Mdr1p are properly localized in *age3*Δ cells

From the hypothesis described above, we expected that, as a consequence of inefficient transport of drug efflux pumps to the membrane, a high amount of these transporters may be present in the cytoplasm instead of the plasma membrane in *age3*Δ cells. To test this hypothesis, we constructed *C. albicans* strains expressing chimeric proteins composed of drug efflux pumps C-terminally fused with GFP. The efflux pumps chosen were Cdr1p [62] and Mdr1p [63], [64], two well studied drug transporters belonging to two distinct classes of efflux pumps. Both proteins are able to efflux azoles [62], [65].

**Figure 8:**
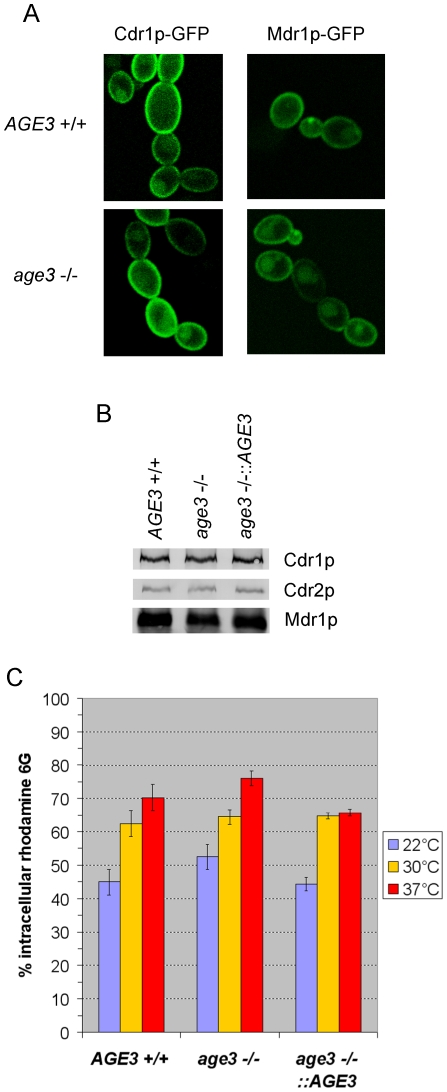
Plasma membrane localization of Cdr1p, Cdr2p and Mdr1p and intracellular rhodamine 6G steady-state level in *age3* mutant cells. (A) Wild-type and *age3*Δ strains which encode either a Cdr1p-GFP fusion protein (TL19 and TL20) or a Mdr1p-GFP fusion protein (TL21 and TL22), respectively, were grown to exponential phase in the presence of doxycycline or benomyl (gene-ON conditions) at 30°C. Cdr1p-GFP and Mdr1p-GFP were visualized by confocal fluorescence microscopy and were predominantly localized in the cytoplasmic membrane independent of the *AGE3* genotype. The diffuse intracellular cloud of GFP fluorescence visible in most cells of all strains colocalizes with the vacuole (DIC images are not shown). (B) The proteins (10 µg per sample) of the membrane fractions, which were isolated from SN87 (*AGE3*+/+), UZ45 (*age3*−/−) and UZ55 (*age3*−/−::*AGE3*) cells each induced either with β-estradiol for *CDR1* and *CDR2* expression or with benomyl for *MDR1* expression, were separated by SDS-PAGE (10%) and blotted onto nitrocellulose membranes. Cdr1, Cdr2 and Mdr1 proteins were detected after immunostaining with polyclonal antibodies (color development with BCIP and NBT). (C) The same strains as in (B) were grown to exponential phase in YPD medium. After washing, the cells were incubated for one hour in 10 mM rhodamine 6G (R6G) in CYG medium at the indicated temperatures. The experiment was done in triplicate and the mean percentage of intracellular R6G level and the standard deviation are shown. At each temperature the intracellular R6G amount in *age3* mutant cells was only slightly higher (<8%) than in wild-type cells.

However, both the GFP fusion protein of Cdr1p and that of Mdr1p are predominantly localized in the plasma membrane in both the *age3*Δ and *AGE3* wild-type background (Fig. 8A). That translocation of drug transporters to the plasma membrane does not significantly depend on Age3p function was further confirmed by Western blotting of plasma membrane proteins isolated from yeast-form cells of the wild-type, the *age3*Δ, and reintegrant strains and immunostaining with antibodies raised against Cdr1p, Cdr2p (which is also able to efflux azoles) and Mdr1p (kindly provided by D. Sanglard) [52]. The results revealed that the plasma membrane of mutant cells contained similar amounts of Cdr1p and Cdr2p and possibly a somewhat lower Mdr1p amount compared to the membranes isolated from wild-type and reintegrant cells (Fig. 8B). Moreover, the intracellular steady-state level of rhodamine 6G (R6G), a substrate of Cdr1p and Cdr2p, but not of Mdr1p [55], is only slightly increased (<8%) in *age3*Δ cells compared to wild-type cells after growth for one hour at 22, 30 or 37°C (Fig. 8C). Furthermore, de-energized (depleted for ATP) exponentially growing *age3* mutant cells showed only a slight increase of R6G influx compared to wild-type cells (data not shown). The R6G efflux rate after glucose-addition (activation of the ATP-dependent efflux pumps) was similar (not shown). These results indicate that the higher drug susceptibility of *age3*Δ cells is not the result of poor transport to the cell membrane and lower activity of the Cdr1 and Cdr2 proteins.

### The *age3*Δ cells are less susceptible to the cell wall-lytic enzyme zymolyase

**Figure 9:**
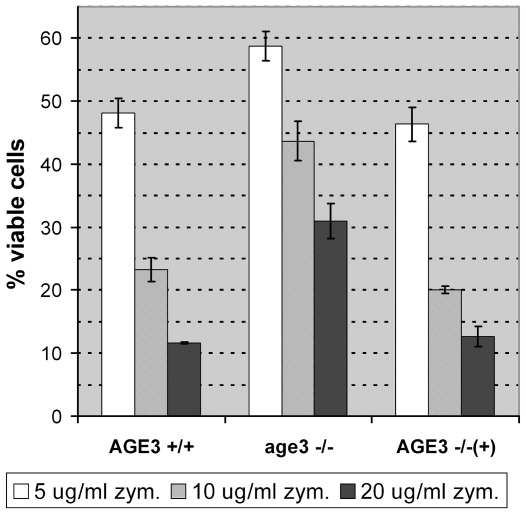
Increased resistance of *age3*Δ cells to zymolyase. The strains SN87 (*AGE3*+/+), UZ45 (*age3*Δ/Δ) and UZ55 (*age3*Δ/Δ::*AGE3*) were grown at 30°C to an OD_600_ of 0.5. An amount of 10^7^ cells per ml was incubated at 37°C for 30 minutes with zymolyase at a concentration of 5, 10 or 20 µg/ml. After addition of an equal volume of 2% SDS the OD_600_ was measured and the percentage of viable cells determined (cells without zymolyase treatment were used as control). The experiment was done in triplicate. The mean percentage and standard deviation are shown. The *age3* mutant cells show a higher resistance to zymolyase compared to wild-type and reintegrant cells.

Since hygromycin B is not a substrate of Cdr1p and Mdr1p [66] and susceptibility of *age3*Δ cells to hygromycin B and SDS points to defects in the cell wall, we investigated the sensitivity of *age3*Δ cells to the cell-wall lytic enzyme zymolyase. Unexpectedly, instead of being more sensitive, the mutant cells were more resistant to zymolyase compared to wild-type cells. This was tested by using two different approaches, cell viability determination (not shown) and measuring the decrease of optical density after spheroplast formation by zymolyase treatment and subsequent cell lysis with SDS (Fig. 9).

## Discussion

In this paper we describe the phenotypic and physiological consequences of deleting the *AGE3* gene from the *C. albicans* genome. Since it is able to complement defects of *S. cerevisiae gcs1*Δ cells, *AGE3* is probably the functional ortholog of the *S. cerevisiae GCS1* gene. The *age3*Δ strain showed a clear delay in endocytosis, which is consistent with an important function of Age3p in endosomal compartments as it has been also shown for its yeast ortholog [29], [33].

### Hyphal and invasive growth defects of the *age3*Δ mutant strain

As we have shown the poor ability of the cells to form true hyphae and the formation of aberrant cell and filament shapes under a number of hyphal growth conditions is possibly caused by the defects in actin polarization. Moreover, the morphology of *age3*Δ cells induced for hyphal growth closely resembles that of other mutants with defective polarization of the actin cytoskeleton [12], [14]. This suggests that, similar to Gcs1p [37], Age3p can stimulate actin polymerization.

Proper formation of the actin cytoskeleton and cortical actin patch localization depends on both exo- and endocytosis [19]. Moreover, cell polarization depends on the localization of the Cdc42 GTPase, the master regulator of polarized growth, to the polar cap. Its essential role in hyphal growth of *C. albicans* is well studied [67]–[69]. It has been shown in yeast that Cdc42p shuttles in a dynamic flux between the cytoplasmic membrane at the polar cap and the cytoplasm [70]. Apart from GDP/GTP recycling on Cdc42p, robust maintenance of cell polarity depends on an actin-dependent efficient exocytic transport of internalized Cdc42p-containing vesicles and subsequent fusion with the cytoplasmic membrane [70]. As discussed above, *age3*Δ cells have a defect in the endocytic pathway. Thus the defect of *age3*Δ cells to maintain the balance of polarized versus isotropic growth, which is required to sustain true hyphal growth after germ tube formation, is probably a combination of disturbed polarization of the actin cytoskeleton and inefficient vesicle trafficking.

As observed for a *C. albicans* strain that lacks the gene encoding for the polarisome component Spa2p [14], the defect of the *age3*Δ strain in hyphal growth was more pronounced on solid than in liquid media. Furthermore, we have shown that *AGE3* is essential for invasive filamentous growth *in vitro*. It is commonly acknowledged that, apart from filamentous growth per se, the ability of *C. albicans* to invade host tissues strongly contributes to virulence of this and other pathogenic fungi [4], [69], [71]. Consistent with this, Epp *et al.* [38] found that the *age3*Δ strain is much less virulent compared to the wild-type strain.

### *AGE3* is required for tolerance of several unrelated toxic compounds including azole drugs

The high susceptibility of *age3* mutant cells to several structurally unrelated toxic compounds, which affect distinct functions of the cell, could be caused simply by the pleiotropic consequences of disturbed vesicle traffic of several routes to different compartments of *age3*Δ cells. Since the drug transporters Cdr1p, Cdr2p and Mdr1p were efficiently transported to the cell membrane in *age3*Δ cells and the steady-state level of rhodamine 6G was in a similar range as in wild-type cells, the high azole sensitivity of mutant cells is probably not the consequence of poor drug efflux activity. In addition, *age3*Δ cells did not show a higher susceptibility to amphotericin B which directly binds to ergosterol [19]. This strongly suggests that *age3*Δ cells have no defect in ergosterol biosynthesis, a process that is targeted by azoles. Taken together, other cellular functions that are involved in tolerance to azoles and possibly also to the other compounds affecting growth of *age3*Δ cells must be compromised in *age3*Δ cells.

Few studies revealed an important role of the vacuole in azole detoxification. *C. albicans* mutant cells lacking the genes of the vacuolar protein sorting factors Vps1p, Vps28p or Vps32p (Snf7p) are more susceptible to fluconazole compared to wild-type cells [72], [73]. Epp *et al.* [38] found that fluconazole exerts a very strong fungistatic activity on *C. albicans* mutant cells lacking the Vma10 subunit of the vacuolar membrane H^+^-ATPase, which they also found for *S. cerevisiae* mutants lacking other V-ATPase subunits. Moreover, several fluconazole-resistant strains isolated from patients after fluconazole administration for several weeks highly accumulate the drug (or some modified form or degradation product) in vesicular vacuoles [74]. Altogether these studies strongly indicate that the vacuole has some important, but yet undefined function in detoxification of azoles.

Interestingly, the *vps1*, *vps28*, *vps32* deletion strains [72], [73] and the fluconazole-resistant strains described by Maebashi *et al.* [74] do not show an increased susceptibility to amphotericin B and 5-fluorocytosine compared to the wild type. This susceptibility pattern agrees with the one we found for *age3*Δ cells. Moreover, we found a higher susceptibility of *age3*Δ cells to hygromycin B, SDS and cadmium ions. In *C. albicans* or *S. cerevisiae* an involvement of the vacuole in susceptibility and/or detoxification of hygromycin B [75], SDS [73] and cadmium [76] has been found. From this compound susceptibility pattern and the endocytic defect of *age3*Δ cells we conclude that the drug susceptibility of *age3*Δ cells may be caused by some yet undefined defect in vacuolar function. A study to confirm this hypothesis is under way. Whether the glutathione *S*-conjugate pump Ycf1p [76] or another glutathione-dependent (or -independent) ABC transporter in the vacuolar membrane, like Mlt1p [77], is able to sequester azoles into the vacuolar lumen is unknown yet. Interestingly, it has been shown that glutathione *S*-transferases, which catalyse the conjugation of glutathione to xenobiotics and other substrates, are involved in fluconazole detoxification in *Schizosaccharomyces pombe* [78].

An involvement in vesicle and protein traffic from the Golgi apparatus to the vacuole via the endosomal route has been shown for yeast Gcs1p [31], [32], [36], [79]. The strong delay of *C. albicans age3*Δ cells in the endocytic route to the vacuole could result in a much lower amount of drug transporters in the vacuolar membrane and toxic compound-degrading enzymes in the vacuolar lumen. This would have a strong impact on susceptibility of the cell to toxic compounds.

The high sensitivity to the cell-wall perturbing compounds hygromycin B and SDS and the reduced sensitivity to cell wall degradation by Zymolyase™ (which includes several enzymes that are able to hydrolyse the major components of the fungal cell wall) compared to the wild type indicate that the cell wall composition of *age3*Δ and wild-type cells differ.

In summary, we conclude that the mutant phenotypes of *age3*Δ cells result from a defect in proper polarization of the actin cytoskeleton, inefficient endocytosis, and possibly inefficient vacuolar functions. The phenotypic consequences of the absence of Age3p described here probably have strong impacts on virulence of *C. albicans* and antifungal therapy which both are affected by deletion of *AGE3* [38]. Therefore, the protein is an ideal candidate target for the development of new antifungal compounds.

## Supporting Information

Figure S1 Southern blots confirming the AGE3 genomic regions of the homozygous age3 mutant (A) and AGE3-reconstituted strains (B). Genomic DNA isolated from the strains indicated was digested with restriction enzymes and the fragments separated by agarose gel electrophoresis. After blotting onto a nylon membrane and hybridization with a [32P]-labelled probe, AGE3 promoter-specific fragments were visualized by phosphoimaging. DNA was digested either with SnaBI and KpnI (A) or with EcoRV and XbaI (B). For both the homozygous age3 mutant and the AGE3-reconstituted strains the expected fragment patterns were observed. (*) For the reconstituted strain two AGE3 promoter-specific fragments (ca. 1620 and 5000 bp) were expected.
(0.78 MB TIF)

Figure S2 Multistep construction of the pTet-GFP plasmid. Restriction sites shown in parentheses are not singular on the corresponding plasmid, all other sites shown are singular. * At this XbaI site, only DNA isolated from E. coli dam- strains can be cut. The final construct is composed of the gene cassettes and fragments shown in the table at the right side cloned on a pBluescript KS(+) backbone. The generation of the inserted PCR products of each cloning step is shown in the table. pTet-GFP can be used for construction of C-terminal gene fusions with GFP. The gene of interest should be inserted between the singular EcoRI (or alternatively, XhoI, SalI, PstI or SmaI) and NgoMIV (NaeI) sites. The translational start codon of the gene has to be included immediately following the EcoRI site. Upstream of the NgoMIV site three spacer tandem repeats of Gly-Ala codons (which separate the fused genes) should be inserted at the end of the gene without a stop codon. The resulting recombinant plsamid can be linearized in the RPS1' gene fragment using the singular restriction sites AgeI or BglII (not shown) and integrated into the RPS1 gene. After transformation, recombinant C. albicans clones can be selected for hygromycin B resistance. The chimaeric gene is under control of the Tet promoter and can be induced by addition of doxycycline to the growth medium. Alternatively, if the plasmid is to be integrated into the native gene locus (ORF), the expression of the GFP fusion construct will be controlled by the native gene promoter.
(1.30 MB TIF)

Table S1 Oligonucleotides used in this study.
(0.06 MB DOC)

Table S2 Plasmids used in this study.
(0.04 MB DOC)
