# Supplementary figures and images for: Candida albicans AGE3, the Ortholog of the S. cerevisiae ARF-GAP-Encoding Gene GCS1, Is Required for Hyphal Growth and Drug Resistance
Source: PLoS One. 2010 Aug 5;5(8):e11993. doi: 10.1371/journal.pone.0011993 (PMC2916835; doi:10.1371/journal.pone.0011993)

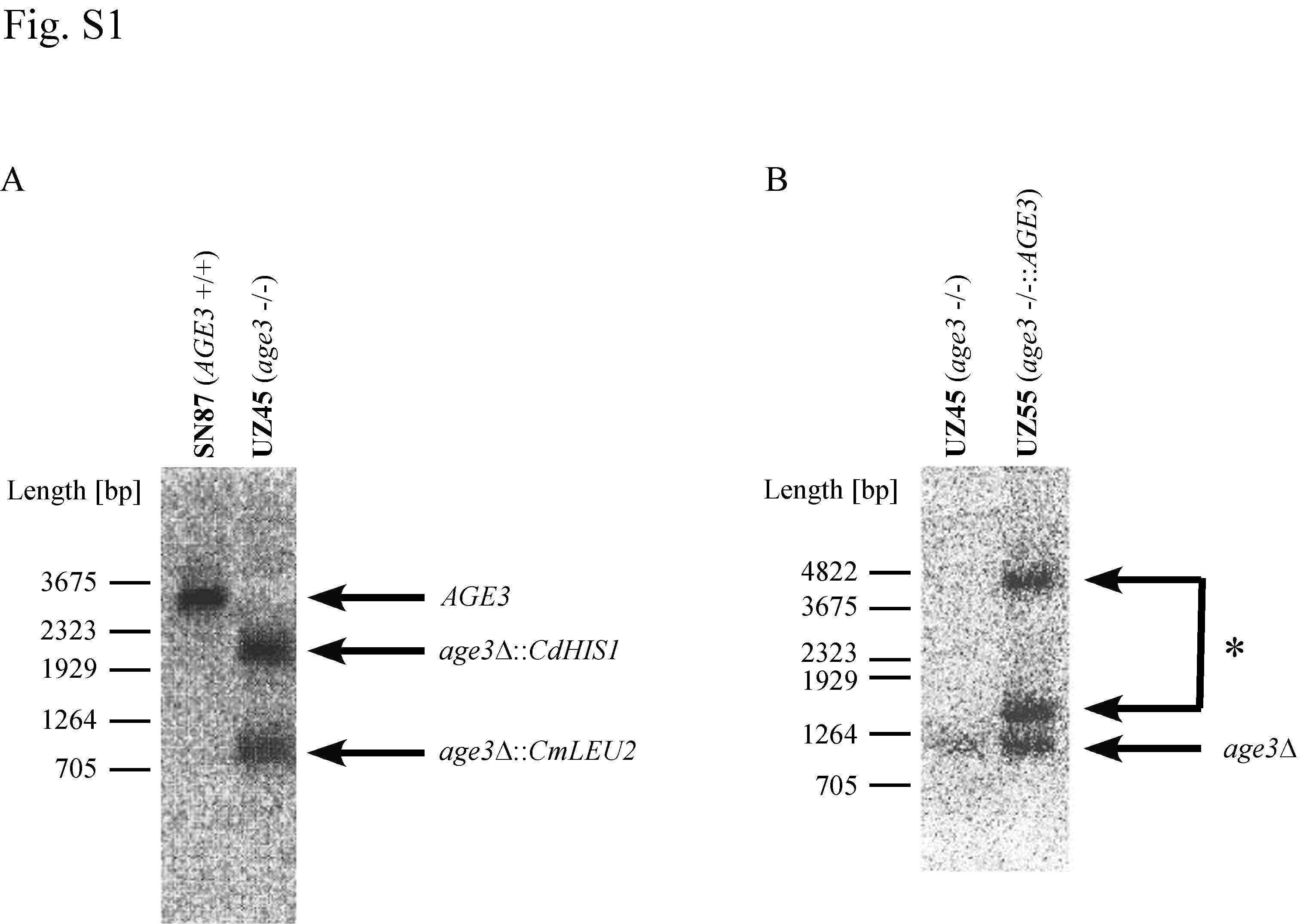

Supplement: Figure S1 — Southern blots confirming the AGE3 genomic regions of the homozygous age3 mutant (A) and AGE3-reconstituted strains (B). Genomic DNA isolated from the strains indicated was digested with restriction enzymes and the fragments separated by agarose gel electrophoresis. After blotting onto a nylon membrane and hybridization with a [32P]-labelled probe, AGE3 promoter-specific fragments were visualized by phosphoimaging. DNA was digested either with SnaBI and KpnI (A) or with EcoRV and XbaI (B). For both the homozygous age3 mutant and the AGE3-reconstituted strains the expected fragment patterns were observed. (*) For the reconstituted strain two AGE3 promoter-specific fragments (ca. 1620 and 5000 bp) were expected. (0.78 MB TIF) [file pone.0011993.s001.tif]

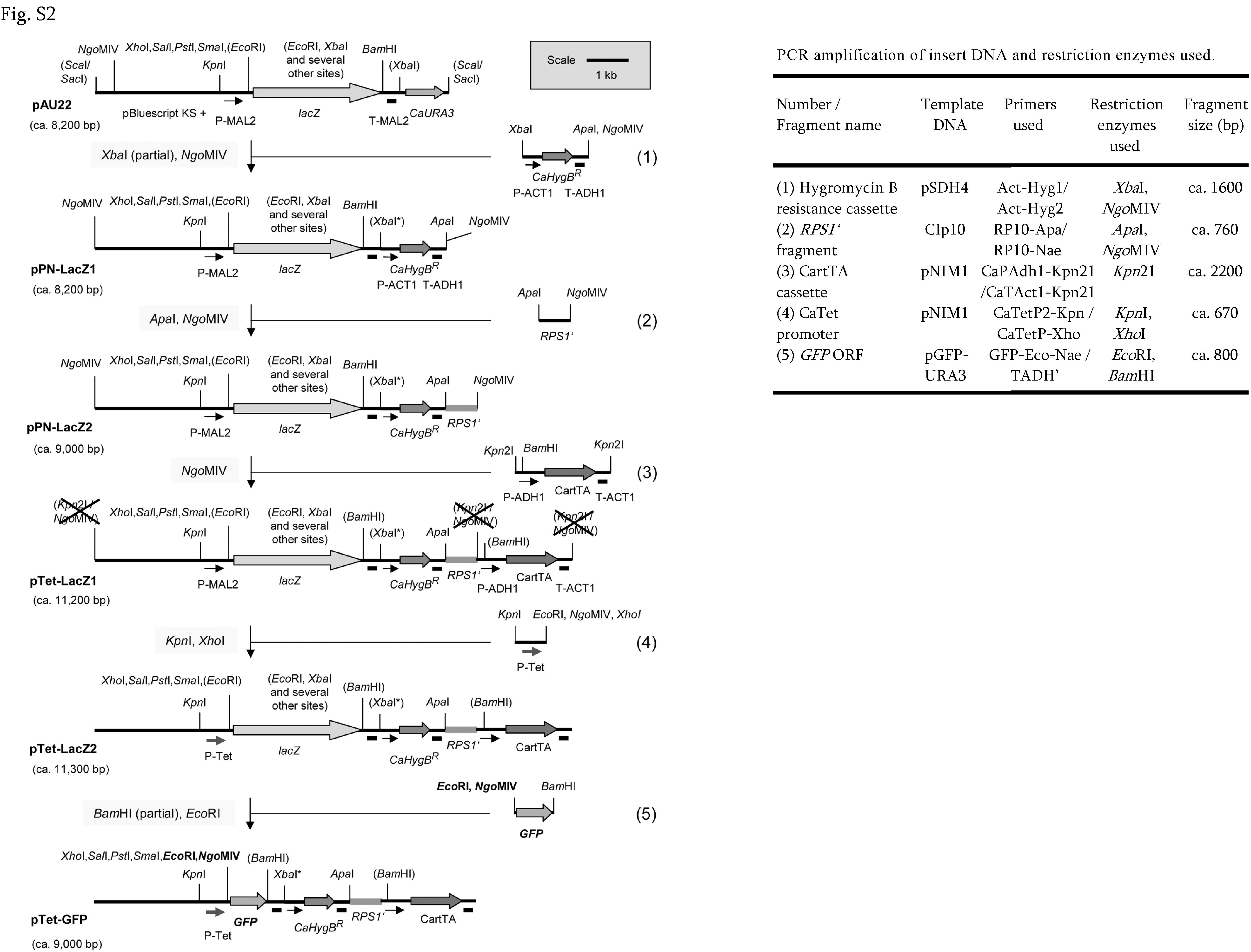

Supplement: Figure S2 — Multistep construction of the pTet-GFP plasmid. Restriction sites shown in parentheses are not singular on the corresponding plasmid, all other sites shown are singular. * At this XbaI site, only DNA isolated from E. coli dam- strains can be cut. The final construct is composed of the gene cassettes and fragments shown in the table at the right side cloned on a pBluescript KS(+) backbone. The generation of the inserted PCR products of each cloning step is shown in the table. pTet-GFP can be used for construction of C-terminal gene fusions with GFP. The gene of interest should be inserted between the singular EcoRI (or alternatively, XhoI, SalI, PstI or SmaI) and NgoMIV (NaeI) sites. The translational start codon of the gene has to be included immediately following the EcoRI site. Upstream of the NgoMIV site three spacer tandem repeats of Gly-Ala codons (which separate the fused genes) should be inserted at the end of the gene without a stop codon. The resulting recombinant plsamid can be linearized in the RPS1' gene fragment using the singular restriction sites AgeI or BglII (not shown) and integrated into the RPS1 gene. After transformation, recombinant C. albicans clones can be selected for hygromycin B resistance. The chimaeric gene is under control of the Tet promoter and can be induced by addition of doxycycline to the growth medium. Alternatively, if the plasmid is to be integrated into the native gene locus (ORF), the expression of the GFP fusion construct will be controlled by the native gene promoter. (1.30 MB TIF) [file pone.0011993.s002.tif]
