# Supplementary material for: Candida albicans AGE3, the Ortholog of the S. cerevisiae ARF-GAP-Encoding Gene GCS1, Is Required for Hyphal Growth and Drug Resistance
Source: PLoS One. 2010 Aug 5;5(8):e11993. doi: 10.1371/journal.pone.0011993 (PMC2916835; doi:10.1371/journal.pone.0011993)
Supplement: Table S1 — Oligonucleotides used in this study. (0.06 MB DOC) [file pone.0011993.s003.doc]

**Table S1**. Oligonucleotides used in this study.

| **Name** | **Features / Application** | **5´-3´ Sequence** |
| --- | --- | --- |
| ACT-Hyg1 | PCR-amplification of *CaHygB*^R^; construction of pPN-LacZ1 | ccagttctagatccagcgtcaaaactagag |
| ACT-Hyg2 | PCR-amplification of *CaHygB*^R^; construction of pPN-LacZ1 | gatcagccggcatgctgggcccagaggtaaacccagaaagca |
| CA1145-2 | Amplification of *AGE3* gene (incl its native promoter and terminator sequence; for cloning of the gene in pCaAge3-Sat2 | gaccttcccgggtcgtgcaatgtacaaga |
| CaAge3-3 | Sequencing of *AGE3* | gatatatcacttggagaataga |
| CaAge3-4 | Sequencing of *AGE3*; *AGE3* probe amplification | ccaaaagcatttggaacttga |
| CaAge3-5 | Sequencing of *AGE3*; *AGE3* probe amplification | gtgttgatttaaatttatcacca |
| CaAge3-6 | Sequencing of *AGE3* | ccttcttatcatgtccattga |
| CaAge3-7 | Amplification of *AGE3* ORF for construction of pCaAge3-2; binds at the *AGE3* start codon | gaccggatccaactatgtccattgatccagaaact |
| CaAge3-8 | Amplification of *AGE3* ORF for construction of pCaAge3-2; binds at the end of the *AGE3* ORF | gaccggatcctcattaaaagtcatcccatttatcat |
| CaAge3-9 | Amplification and cloning of the *AGE3* gene (incl. its native promoter); binds upstream of the *AGE3* ORF | gaccttctgcaggtcgacgatcttttaataccgttaaagct |
| CaAge3-d1 | Amplification of *AGE3* upstream region for construction of deletion cassette | gccattaatgctccaaaaggtggt |
| CaAge3-d3 | Amplification of *AGE3* upstream region for construction of deletion cassette | cacggcgcgcctagcagcggataagaaggataaaggatcaat |
| CaAge3-d4 | Amplification of *AGE3* downstream region for construction of deletion cassette | gtcagcggccgcatccctgctggtggatcaaagaaaaacga |
| CaAge3-d6 | Amplification of *AGE3* downstream region for construction of deletion cassette | gatatatcacttggagaataga |
| CaCdr1-Ngo | PCR-amplification of *CDR1* ORF; construction of pCdr1-GFP | gatcagccggctttcttattttttttctctctgttac |
| CaCdr1-Sal | PCR-amplification of *CDR1* ORF; construction of pCdr1-GFP | gatcagtcgacatgtcagattctaagatgtcgtc |
| CaHygB-3 | PCR verification of *C.albicans* clones with integrated plasmids pAge3-GFP, pCdr1-GFP and pMdr1-GFP | ctcaaggtgttactttgcaaga |
| CaHygB-4 | PCR verification of *C.albicans* clones with integrated plasmids pAge3-GFP, pCdr1-GFP and pMdr1-GFP | cttggagaaccagccaattct |
| CaMdr1-Eco | PCR-amplification of *MDR1* ORF; construction of pMdr1-GFP | gatcagaattcatgcattacagatttttgagaga |
| CaMdr1-Ngo | PCR-amplification of *MDR1* ORF; construction of pMdr1-GFP | gatcagccggcattagcatacttagatcttgctc |
| CaPAdh1-Kpn2I | PCR-amplification of *CartTA* gene cassette; construction of pTet-LacZ1 | cagtctccggaccgcggttgagatggagcc |
| CaTAct1-Kpn2I | PCR-amplification of *CartTA* gene cassette; construction of pTet-LacZ1 | cagtctccggagcagacattttatgatggaatga |
| CaTetP2-Kpn | PCR-amplification of CaTet promoter; construction of pTet-LacZ2 | cttacggtacccacagtttggttcagcacct |
| CaTetP-Xho | PCR-amplification of CaTet promoter; construction of pTet-LacZ2 | gaactcgagtatttatatttgtatgtgtgtagga |
| CaUniv-2 | Amplification of *CdHIS1* and *CmLEU2* genes for construction of *AGE3* deletion cassettes | ccgctgctaggcgcgccgtgaccagtgtgatggatatctgc |
| CaUniv-5 | Amplification of *CdHIS1* and *CmLEU2* genes for construction of *AGE3* deletion cassettes | gcagggatgcggccgctgacagctcggatccactagtaacg |
| CdHis1-1 | Verification of correct integration of deletion cassette | gtcaatcatttgcccgatcgt |
| CdHis1-2 | Verification of correct integration of deletion cassette | atgttgtcgaattggtctgct |
| CdHis1-3 | Verification of correct integration of plasmids into *AGE3* reintegrant strain | cctcacacaacaatccatgt |
| CdHis1-4 | Verification of correct integration of plasmids into *AGE3* reintegrant strain | gacgttcaacttgcgtacct |
| CmLeu2-1 | Verification of correct integration of deletion cassette | gcaattgaagctgccactcct |
| CmLeu2-2 | Verification of correct integration of deletion cassette | ccaataaggagtcactggca |
| CmLeu2-3 | Verification of correct integration of plasmids into *AGE3* reintegrant strain | gcttcaccaaacccagcta |
| CmLeu2-4 | Verification of correct integration of plasmids into *AGE3* reintegrant strain | ctctcaccggtttacttgga |
| GFP-Eco-Nae | PCR-amplification of *GFP* ORF; construction of pTet-GFP | gcatgaattcatgggtgccggcgctggtgcatctaaaggtgaagaattattcact |
| RP10-Apa | PCR-amplification of *RPS1’* fragment; construction of pPN-LacZ2 | ccagtgggcccaactcaagtacaacatgg |
| RP10-Nae | PCR-amplification of *RPS1’* fragment; construction of pPN-LacZ2 | ccgtagccggcaaagccaataatgaacccca |
| TADH` | PCR-amplification of *GFP* ORF; construction of pTet-GFP | aagatcacgcgtctcgaga |
