# Supplementary material for: Candida albicans AGE3, the Ortholog of the S. cerevisiae ARF-GAP-Encoding Gene GCS1, Is Required for Hyphal Growth and Drug Resistance
Source: PLoS One. 2010 Aug 5;5(8):e11993. doi: 10.1371/journal.pone.0011993 (PMC2916835; doi:10.1371/journal.pone.0011993)
Supplement: Table S2 — Plasmids used in this study. (0.04 MB DOC) [file pone.0011993.s004.doc]

**Table S2**. Plasmids used in this study.

| **Name** | **Description** | **Source, reference** |
| --- | --- | --- |
| CIp10 | Carries a fragment of the *C. albicans* *RPS1* gene. Integration into one allele has no phenotypic consequences. Cip10 was used for construction of pTet-GFP (Fig. S2). | [46] |
| pAU22 | Starting plasmid for construction of pTet-GFP (Fig. S2). | [45] |
| pCaAct-Sat1 | [P_ACT1_-*SAT1*-T_ADH1_] cassette in YCplac33; used for construction of pCaAge3-Sat2 | This study |
| pCaAge3-2 | *AGE3* ORF cloned behind inducible *S. cerevisiae* Tet-promoter on plasmid pCM252-U. | This study |
| pCaAge3-Sat2 | Carries the *AGE3* gene including 720 bp upstrem of the start codon; used for reintegration of *AGE3* into the *AGE3* promoter region of the *age3*Δ/Δ strain*.* | This study |
| pCdr1-GFP | Encodes for the C-terminal fusion of GFP to Cdr1p. | This study |
| pCM252 | Yeast centromeric plasmid with inducible Tet-promoter system; carries *TRP1* marker gene. | [44] |
| pCM252-U | Yeast centromeric plasmid with inducible Tet-promoter system; carries *URA3* marker gene. | This study |
| pGFP-URA3 | Carries the *GFP* gene used for the construction of pTet-GFP (Fig. S2). | [48] |
| pMdr1-GFP | Encodes for the C-terminal fusion of GFP to Mdr1p. | This study |
| pNIM1 | Carries the *SAT1* gene conferring nourseothricin resistance and the *C. albicans* Tet promoter system inducible by doxycycline; used for construction of pTet-GFP and pCaAge3-Sat2 (gene cassette for reintegration of the *AGE3* gene into the *age3*Δ strain). | [49] |
| pPN-LacZ1 | Intermediate product for the construction of pTet-GFP (Fig. S2). | This study |
| pPN-LacZ2 | Intermediate product for the construction of pTet-GFP (Fig. S2). | This study |
| pSDH4 | Carries the *C. albicans* codon-optimized hygromycin B resistance gene which was derived from pAU34-HygB^R^ (81); used in construction of pTet-GFP (Fig. S2). | Bito A., unpublished |
| pSDS4 | Carries [P_ACT1_-*SAT1*-T_ADH1_] gene cassette derived from pSFS1A (62); used for construction of pCaAct-Sat1 | Bito A., unpublished |
| pSN40 | Template for amplification of the *C. maltosa* *LEU2* gene (*CmLEU2*); the resulting PCR product was used subsequently for creating the *AGE3* deletion cassette by fusion PCR. | [47] |
| pSN52 | Template for amplification of the *C. dubliniensis HIS1* gene (*CdHIS1*); the resulting PCR product was used subsequently for creating the *AGE3* deletion cassette by fusion PCR. | [47] |
| pTet-GFP | Plasmid for constructing C-terminal gene fusions with GFP (Fig. S2). | This study |
| pTet-LacZ1 | Intermediate product for the construction of pTet-GFP (Fig. S2). | This study |
| pTet-LacZ2 | Intermediate product for the construction of pTet-GFP (Fig. S2). | This study |
| YCplac33 | Yeast centromeric plasmid with *URA3* marker gene | [43] |
